# Surgeon’s Experience May Circumvent Operative Volume in Improving Early Outcomes After Pancreaticoduodenectomy

**DOI:** 10.7759/cureus.42927

**Published:** 2023-08-03

**Authors:** Muhammad A Khan, Shah Muhammad, Haider Mehdi, Abida Parveen, Uzma Soomro, Jehangir Farman Ali, Abdaal W Khan

**Affiliations:** 1 Hepato-Pancreato-Biliary (HPB) Surgery, Sindh Institute of Urology and Transplantation, Karachi, PAK; 2 General Surgery, Sindh Institute of Medical Sciences, Karachi, PAK; 3 Transplant Surgery, Sindh Institute of Urology and Transplantation, Karachi, PAK

**Keywords:** delayed gastric emptying, pancreaticoduodenectomy, pylorus-preserving pancreaticoduodenectomy, pancreaticoduodenectomy (pd), overall survival (os), short-term outcome, complications, low-volume center, postoperative pancreatic fistula

## Abstract

Introduction

Pancreaticoduodenectomy (PD) is a complex procedure with a significant proportion of postoperative complications and improving but notable mortality. PD was the prototype procedure that initiated the lingering debate about the relationship of better operative outcomes when performed at higher-volume centers. This has not translated into practice. Impediments include the absence of a universally accepted definition of a high-volume center among others. Contrary evidence suggests equivalent outcomes for PD at low-volume centers when performed by experienced hepatobiliary surgeons. We reviewed our perioperative outcomes for PD from an earlier period as a low-volume center with an experienced team.

Methods

A longitudinal study of all PDs completed in our department between 2012 and 2017 was performed.

Results

A total of 28 PD were performed during this period. Pylorus-preserving PD was performed in 23 patients and classical PD in the remaining. A separate Roux-en-Y loop was used for high-risk pancreatic anastomosis in six cases. The mean patient age was 49.3±12.4 years. The male-to-female ratio was 1.3:1. Preoperative drainage procedures were carried out in 19 patients. The mean serum total bilirubin level was 3.98(±4.5) mg/dL. There was no 90-day mortality. Postoperative complications included wound infection in 10 (36.7%) and respiratory complications in 10 (36.7%) patients. Postoperative bleeding requiring intervention occurred in one patient, and two patients had an anastomotic leak (one pancreatojejunostomy (PJ) and one gastrojejunostomy (GJ)). Delayed gastric emptying (DGE) was noted in three (10.7%) patients. The mean length of hospital stay was 14±7 days. The median overall survival (OS) was 84 months.

Conclusion

Comparable early outcomes can be achieved at low-volume centers for patients undergoing PD with an experienced team, optimal patient selection, and the ability to rescue for complications.

## Introduction

Pancreaticoduodenectomy (PD) is veritably considered among the most complex abdominal operative procedures. The reputation has lasted more than 85 years since the procedure was described by Whipple et al. in 1935 [1]. This is related in large measure to the 6-8 hours of duration of the operation, a reported postoperative complication rate of 30%-60%, and perioperative mortality of 1.5%-3% [2-7]. Following a review of statewide outcome data from New York in 1995, spanning six years, it was demonstrated that the outcome of the procedure significantly improved when performed at a high-volume compared to a low-volume center [8]. With endorsement from a number of studies, there has been a deliberation that the procedure should be centralized to larger-volume institutions [6,9-12]. This impetus has not unequivocally translated to practice, and a substantial proportion of cases continue to be performed at low-volume centers [6,11]. Part of the problem is the absence of a widely agreed definition of high- and low-volume centers. The Leapfrog criteria, for example, recommends a hospital volume of 20 pancreatic procedures and 10 cases per surgeon every year for improving outcomes [9]. When a statewide study attempted to classify high- and low-volume centers for outcomes comparison of PD using Leapfrog criteria, none of the centers qualified as a high-volume center among the 157 centers included over a five-year period [6]. Their analysis revealed patient’s reluctance in traveling to distant centers with associated racial, financial, age, and education-related factors as significant barriers. Similar regional dynamics with added limitations of available facilities likely pervade around the world, limiting the pragmatic application of this concept. Additionally, increasing evidence suggests that the premise may not hold true if experienced surgeons perform the procedure [12-15], and their volume of related hepatobiliary procedures may augment their outcome for PD [16]. We reviewed outcomes of PD procedures performed in an earlier period as a dedicated hepato-pancreato-biliary (HPB) surgical unit with low volume (less than 10/year). We intended to determine if our cumulative prior operative experience provided an adequate safeguard for optimal patient outcomes despite a low case volume.

## Materials and methods

A retrospective longitudinal study of all PDs done in our department from January 2012 to December 2017 was performed, after the detailed evaluation of the research proposal by the Ethical Review Committee of Sindh Institute of Urology and Transplantation (SIUT-ERC) and obtaining approval (SIUT-ERC-2022/A-373). A total of 28 patients are included in the study. Our patient selection for PD was conservative, and operative resection was offered to patients with favorable operative risk and resectable disease. Operative risk assessment involved evaluating the patient’s Eastern Cooperative Oncology Group (ECOG) status, comorbidities, ability to perform effective incentive spirometry (500 mL × 10 times) on repeated assessment, and completion of a simplified 100-feet walk test. Although this study is focused on early postoperative outcome of PD as a procedure rather than a specific indication of PD, for instance, adenocarcinoma of the ampulla or head of the pancreas, the general selection criteria in cases with malignancy was again locally confined disease with no vascular involvement. In large measure, this was in view of the poor prognosis in the absence of effective adjuvant therapy at the time.

Most patients underwent pylorus-preserving PD. In select cases, conventional Whipple’s resection was performed, where concerns arose about the vascularity of the duodenal stump following resection or when our operative assessment indicated that the mass was situated too close to the duodenal resection margin. Furthermore, the duct-to-mucosa pancreatojejunostomy (PJ) technique was performed with 6/0 polydioxanone sutures (PDS) in an end-to-side fashion. A separate Roux loop for PJ was sometimes used for high-risk cases (soft gland, <2 mm duct). Hepatojejunostomy (HJ) was performed 10 cm distal to PJ with 4/0 or 5/0 PDS. Gastrojejunostomy (GJ) or duodenojejunostomy (DJ) was performed 45 cm distal to HJ in a retrocolic fashion. During this phase, our PD procedures were performed by a team of four surgeons. Two of these surgeons had prior experience exceeding 30 individually performed pancreatic resections at other academic medical centers. Their cumulative operative experience for major abdominal procedures exceeded a thousand cases, for either of them. For each procedure, resection was completed by a team of two surgeons, followed by anastomotic reconstruction by another team of two surgeons, in most cases. We followed a standardized technique in all the cases.

Data was collected regarding demography and perioperative laboratory results, noting any prior interventions such as endoscopic retrograde cholangiopancreatography (ERCP) or percutaneous transhepatic cholangiography (PTC) and operative details such as the duration of surgery, blood loss, blood transfusion, and the type of procedure performed. The surgical quality parameters assessed included completeness of resection (R0, R1, and R2) and the number of nodes resected. The follow-up outcome data collected included respiratory and wound complications, any other complication requiring prolonged hospital stay or additional intervention (either by interventional radiology or surgical), the incidence of delayed gastric emptying (DGE), and any anastomotic leak, including postoperative pancreatic fistula (POPF). POPF was diagnosed and graded based on the 2005 International Study Group of Pancreatic Fistula (ISGPF) criteria [17] since the revised criteria [18] were not available during the period studied. Additionally, perioperative mortality and patient survival were noted.

## Results

**Figure 1:**
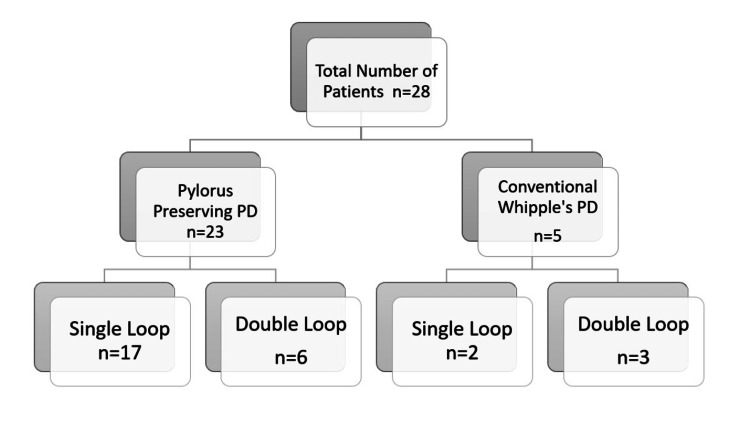
Patient allocation PD: pancreaticoduodenectomy

A total of 28 PD were performed during this period. Pylorus-preserving PD was performed in 23 patients, while classical PD was performed in the remaining patients (Figure 1).

The average age of the patient was around 50 years, with a minimum of 29 and a maximum of 75 years. The male-to-female ratio was 1.3:1. The mean preoperative albumin was 3.3(±0.62). Preoperative drainage procedures were carried out in two-thirds (n=17) of patients in the form of ERCP or PTC. The mean serum total bilirubin level was 3.98(±4.5) mg/dL, with a mean alkaline phosphatase of 332.33(±152.42) units/L. These baseline characteristics are given in Table 1.

**Table 1:** Patients’ baseline characteristics SD: standard deviation, ERCP: endoscopic retrograde cholangiopancreatography, PTC: percutaneous transhepatic cholangiography

Characteristic	Number	%
Total number of patients	28	100
Male	17	56.6
Female	13	43.3
Age (years) (mean±SD (range))	49.3±12.4 (29-75)	
Preoperative drainage		
Not performed	11	36.6
Performed	19	63.3
ERCP	15	50
PTC	3	10
ERCP+PTC	1	3.3
Preoperative laboratory results		
Bilirubin (mg/dL) (mean±SD)	3.98±4.52	
Bilirubin in the non-drainage group (mg/dL) (mean±SD)	3.46±3.87	
Bilirubin in the drainage group (mg/dL) (mean±SD)	4.26±4.96	
Serum alkaline phosphatase (units/L) (mean±SD)	332.33±152.42	
Serum albumin (gm/dL) (mean±SD)	3.05±0.67	
Hemoglobin (gm/dL) (mean±SD)	11.74±1.63	

The mean operative time was 444(±84) minutes. The mean operative blood loss was 373(±248) mL. A double-loop reconstruction was utilized for high-risk pancreatic anastomosis in nine cases.

Histologically, two-thirds of tumors were labeled ampullary (21), and 79% were adenocarcinoma (n=22), well-differentiated (n=14), and moderately differentiated (n=8). R0 resection was achieved in all but one case (R1). The mean tumor size was 29.1(±14.3) mm. The average nodes examined by the pathologist were 7(±3.7), and node-positive disease was found in seven (25%) cases. There was no 90-day mortality, as presented in Table 2.

**Table 2:** Postoperative complications

Complication	Number	%
Surgical site infection	10	36.71
Respiratory	10	36.71
Hemorrhage	1	3.57
Anastomotic leak	2	7.14
Postoperative pancreatic fistula	1	3.57
Delayed gastric emptying	3	10.71
Death	0	0

**Figure 2:**
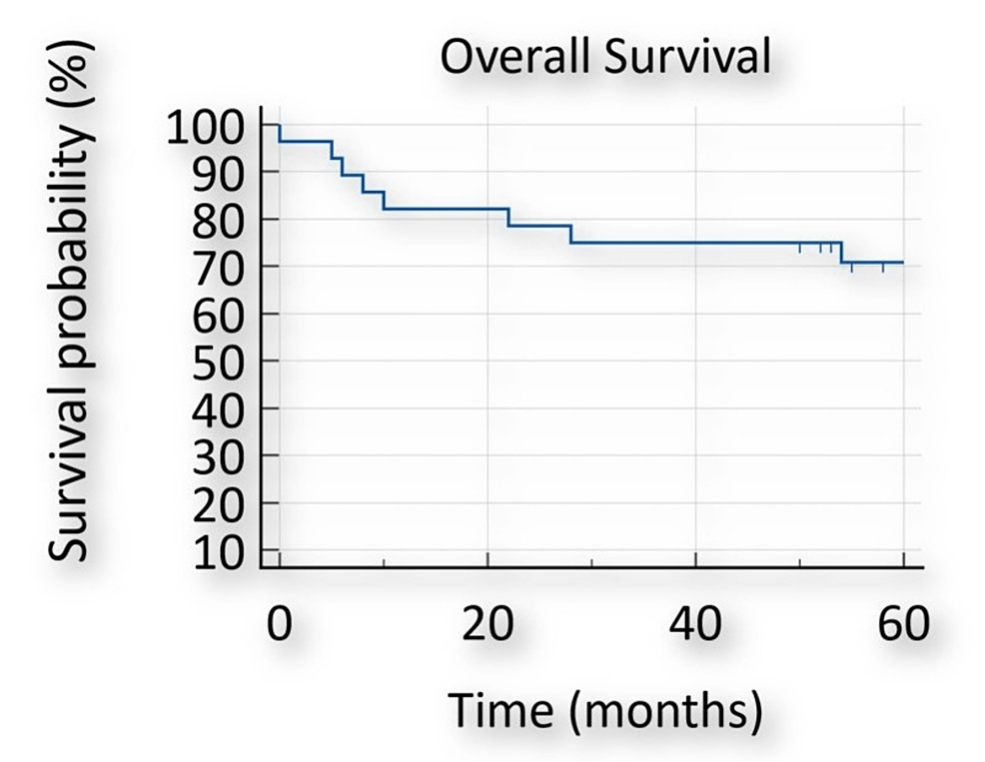
Kaplan-Meier survival curve for overall survival following pancreatoduodenectomy

Postoperative complications (Table 2) included wound infection in 10 (36.7%) patients, respiratory complications in 10 (36.7%) patients (three requiring extended hospital stay and four required short-term supplemental oxygen that did not require extension of length of stay), postoperative bleeding in one patient at the time of the removal of drain who required re-exploration and suture of bleeding point, and anastomotic leak in two patients (one PJ and one GJ). Both were treated conservatively without any consequences. Delayed gastric emptying was noted in three (10.7%) patients. The mean length of hospital stay was 14(±7) days. The median overall survival following PD was 85 months (95% confidence interval (CI): 65-100), based on the Kaplan-Meier survival curve assessment, as demonstrated in Figure 2.

A total of eight (28.57%) patients died over the subsequent five-year follow-up period. Among these, a 75-year-old patient died due to pulmonary complications of COVID-19 after 54 months following PD. All the other mortalities resulted from local recurrence or metastatic spread of pancreatic adenocarcinoma. The median survival among this cohort was 20 months.

## Discussion

Pancreaticoduodenectomy is associated with significant morbidity despite increasing operative volumes and experience worldwide. Conversely, there has been a remarkable improvement in perioperative mortality, from a high reported rate exceeding 20% [19,20] to a low of 1.1%-4.3% at high-volume centers [21,22] and between 3.84% and 12.9% in low-volume centers [15,22-24]. There is a wide range in mortality rates evident among the low-volume centers, the lowest of the spectrum matching high-volume centers, whereas up to threefold higher mortality is seen for others. Our results showed a similar morbidity, and there was no perioperative (up to 90 days postoperatively) mortality seen during the period. Some prior studies have analyzed the possible factors to explain the difference in mortality among centers. A recent study correlating morbidity and mortality profiles of different programs looked specifically into “failure to rescue” from morbidity as consequential to subsequent death [25]. Based on multivariate logistic regression analysis, they concluded that a difference in mortality profile relates mostly to a lower failure to rescue rate from incident major morbidity after PD. For an individual high-volume center, the main cause of death within 90 days of PD was multisystem organ failure (MSOF) secondary to sepsis, aspiration, and hemorrhage, followed by cardiac arrest and pulmonary embolism [21]. A low-volume center with a high mortality rate following PD reported septic shock, massive hemorrhage, and thromboembolism as the commonest cause of death in their series [4]. The low early postoperative mortality in our series is then likely attributable to the low incidence of serious complications such as life-threatening sepsis and hemorrhage in the cohort, which was augmented by our ability to rescue when these occurred. We believe that our cumulative experience of managing patients undergoing major operative procedures, the institutional support structures, being an academic tertiary care institution and a major organ transplantation center, a conservative patient selection, and extended preoperative optimization overall contributed to this optimized outcome. Attention to detail such as patients’ preoperative target-oriented nutritional and functional goals, routine preoperative echocardiogram, and almost religious emphasis on achieving forced inspiratory volume of 700-900 mL on incentive spirometry all likely contributed incrementally.

Postoperative pancreatic fistula (POPF) is the single most dreadful complication of PD and the major cause of postoperative mortality. With the improvement of surgical techniques and the use of fine suture materials, this complication has come down to 10%-15% [16-18]. We have only one intra-abdominal abscess, which we considered a consequence of pancreatic leak. This was managed conservatively without major consequences. The reason for such low leak rates may be related to the operative technique. We have also used a separate Roux loop for high-risk pancreatic anastomosis (PJ). A separate Roux loop is historically noted to prevent leak-related mortality [26]. This is hypothesized to prevent the activation of pancreatic enzymes [19,20] that may lead to impaired anastomotic healing.

Moreover, leak from GJ is not frequently reported and is a rare complication. We had one leak from GJ managed conservatively. This was due, in retrospect, to a longer than 3 cm duodenal stump beyond the pylorus that appeared relatively pale with a light blue hue after anastomosis. Ischemia at the duodenal end likely contributed to the leak.

It is controversial that high bilirubin levels and cholestatic liver enzymes compromise surgical outcomes [21], but our practice is to stent the common bile duct (CBD) in patients with a serum total bilirubin level of >5 mg. Based on this fact, we stented 17 patients before surgery to bring the serum total bilirubin level to <5 mg. This also helped us to improve the nutritional status of many patients before surgery.

Although the mean operative time was longer than the high-volume centers, it did not affect the outcome, and the results are equivalent. Similarly, the mean operative blood loss was less than 100 mL in our experience. We have a high threshold for blood transfusion as it worsens the prognosis as suggested by many studies [22,23].

The most common morbidity that we encountered was wound infection [42%] despite all strict measures toward aseptic techniques and timely use of antibiotics. It was a major reason to delay discharge from the hospital. Evidence has shown that biliary stenting does increase septic complications [24], and this may be the explanation for a higher wound infection rate. Delayed gastric emptying is another troublesome problem, with an incidence of 13%-20% [25,26]. Three of our patients faced this problem (11%). This responded to supportive care with parenteral nutrition, and no endoscopic or surgical intervention was needed in our patients.

The duration of hospital stay is again similar to the centers with high volume due to emphasis on early mobilization and minimizing complications.

Western literature shows that peri-ampullary tumor occurs usually in patients in their sixth decade of life, but our population exhibits these tumors mostly in the fifth decade. This trend has been noted in the Asian population in other studies [14,15]. Our patients were mostly early-stage pancreatic ductal adenocarcinoma (PDAC), with a small proportion of neuroendocrine tumors and other benign pathologies. All the mortalities witnessed during a subsequent five-year period following PD were among the PDAC patients, and the median survival was 20 months among this subset, in keeping with the international trend [27], during the study period.

This study has limitations considering the inherent bias in a retrospective review and the low patient volume. Our inferences may be skewed by the low power of a small sample size. These are unavoidable given that we were attempting to quantify the adversity of our low-volume practice in comparison to the standards set by high-volume centers worldwide. Going forward, we intend to compare this experience with our current experience of significantly higher volumes and determine whether the additional experience and volumes led to better outcomes for our patients. As our patients included all patients undergoing PD irrespective of the indication, we have not highlighted long-term oncological outcomes of, for instance, PDAC cases. Even among the PDAC cases, we had very conservative selection criteria as already described. This would at least partially account for the good overall outcome observed in our cohort.

## Conclusions

Our experience lends credence to the notion that comparable surgical outcomes can be achieved for pancreaticoduodenectomy even in low-volume centers. This may be related to better case selection together with good surgical technique especially for PJ anastomosis likely aided by a team’s ability to rescue the patient when complications occurred.
